# Radiomics and Radiogenomics in Evaluation of Colorectal Cancer Liver Metastasis

**DOI:** 10.3389/fonc.2021.689509

**Published:** 2022-01-07

**Authors:** Yun Wang, Lu-Yao Ma, Xiao-Ping Yin, Bu-Lang Gao

**Affiliations:** CT-MRI Room, Affiliated Hospital of Hebei University, Baoding, China

**Keywords:** colorectal cancer, liver metastasis, radiomics, gene, treatment, prognosis

## Abstract

Colorectal cancer is one common digestive malignancy, and the most common approach of blood metastasis of colorectal cancer is through the portal vein system to the liver. Early detection and treatment of liver metastasis is the key to improving the prognosis of the patients. Radiomics and radiogenomics use non-invasive methods to evaluate the biological properties of tumors by deeply mining the texture features of images and quantifying the heterogeneity of metastatic tumors. Radiomics and radiogenomics have been applied widely in the detection, treatment, and prognostic evaluation of colorectal cancer liver metastases. Based on the imaging features of the liver, this paper reviews the current application of radiomics and radiogenomics in the diagnosis, treatment, monitor of disease progression, and prognosis of patients with colorectal cancer liver metastases.

## 1 Introduction

Colorectal cancer (CRC) is the third most prevalent malignancy and the second commonest cause of cancer-related deaths throughout the world (1), with the incidence and mortality still on the rise in recent years (2). Because of the hepatic unique blood circulation characteristics, the liver has become the most common organ for blood metastasis of cancers, accounting for 25% of all cancer metastasis (3) and approximately 35%–55% of CRC (4, 5). The liver has uniquely favorable conditions for stagnation and growth of cancerous cells, with double blood supply from the visceral and portal vascular systems and natural spaces among adjacent endothelial sinusoidal cells that are deficient of a typical basement membrane for covering (3, 6, 7). Hepatic metastasis is a critical indicator of prognosis for patients with primary cancers, and the life expectancy of patients with hepatic metastases from gastrointestinal cancers is only 6 months without appropriate treatment (8). Accurate prediction and differentiation of liver metastases from CRC is critical to making an appropriate therapeutic plan and improving the prognosis of the patients. Ultrasound, computed tomography (CT), magnetic resonance imaging (MRI), and positron emission tomography (PET) have been routinely applied to detect and assess liver lesions, including metastases of cancer (9, 10). Some liver metastatic lesions from primary cancers of different systems may have common characteristics, including hyperechoic lesions surrounded by a hypoechoic halo (targeted ring sign) in primary gastrointestinal and vascular carcinomas on ultrasound imaging and presence of calcification in CRC or ovarian carcinomas (7, 11, 12). Metastatic lesions with typical imaging features may be easily identified from specific primary carcinomas; however, this kind of lesion accounts for only a small proportion of metastatic lesions, with most of the metastatic lesions being atypical on imaging, whose specific origin cannot be identified easily. Thus, thorough laboratory and physical examinations, molecular genetic test, and tissue biopsy have been applied to assess the primary origin of liver metastases even though these tests and examinations are costly, invasive, or time-consuming (13, 14).

**Figure 1:**
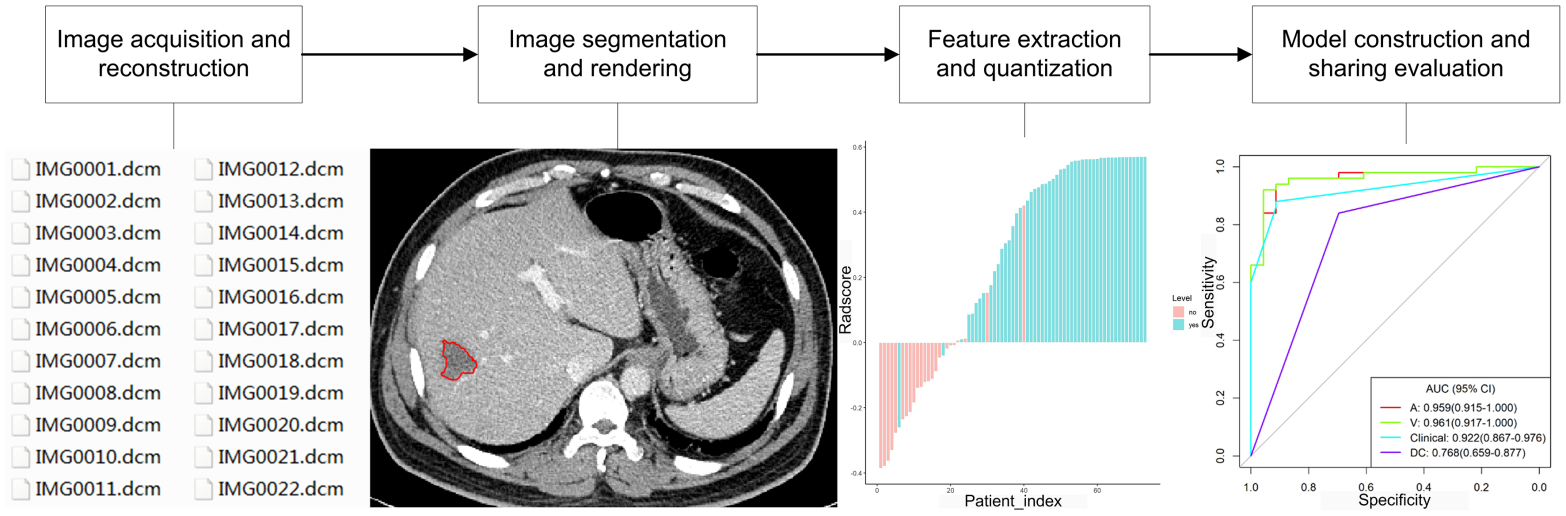
Framework of radiomics application.

With the development of great-volume computing capability, it is currently feasible to quickly extract countless quantitative characteristics from three-dimensional imaging data of MRI, CT, ultrasound, and PET for evaluation of the nature of different lesions, because digital medical images contain considerable information that reflects potential pathophysiology. This technology of transforming digital medical imaging data into high-dimensional data for assessment and decision support is referred to as radiomics (15). The framework of radiomics application is shown in **Figure 1**. The radiomics technology has been motivated by the notion that biomedical images comprise information that mirrors and can be used to reveal basic pathophysiology through quantitative analysis. It has been applied in many conditions, but the most developed field of application is in oncology. Quantitative features of imaging are based on imaging shape, intensity, volume, size, and texture, which provide detailed information on tumor microenvironment and phenotype distinct from that offered by laboratory results, clinical reports, and genomic or proteomic analyses. Combined with other clinical information, these features can be used for correlation analysis with clinical results and decision-making, and radiomics can thus provide countless imaging biomarkers to potentially help cancer diagnosis, detection, prognosis evaluation, prediction of treatment response, and monitoring of disease progression. Radiomics is a young field of study and will undergo a slow progress because of technical complexity, datum overfitting, deficiency of standards for outcome validation, incomplete presentation of outcomes, and unrecognizable confounding factors in the databases.

Radiogenomics refers to the exploring of radiomics data to find correlations with genomic modes and has aroused considerable interest in the research field of oncology (15). Here, in this paper, radiogenomics only indicates the combination of genomic information and radiomic features to enable decision support rather than whole-genome analysis to determine the genetic causes of radiosensitive variations in the scope of radiation oncology. Radiogenomics is important because not all patients have had their cancerous diseases genomically profiled even though they may undergo imaging examinations during the course of disease. Radiogenomic data can provide gene expression or mutation information to increase diagnostic, predictive, and prognostic capability and to enable precision therapy because these radiomics data are originated from the complete tumor lesion rather than a small sample of tissue.

**Figure 2:**
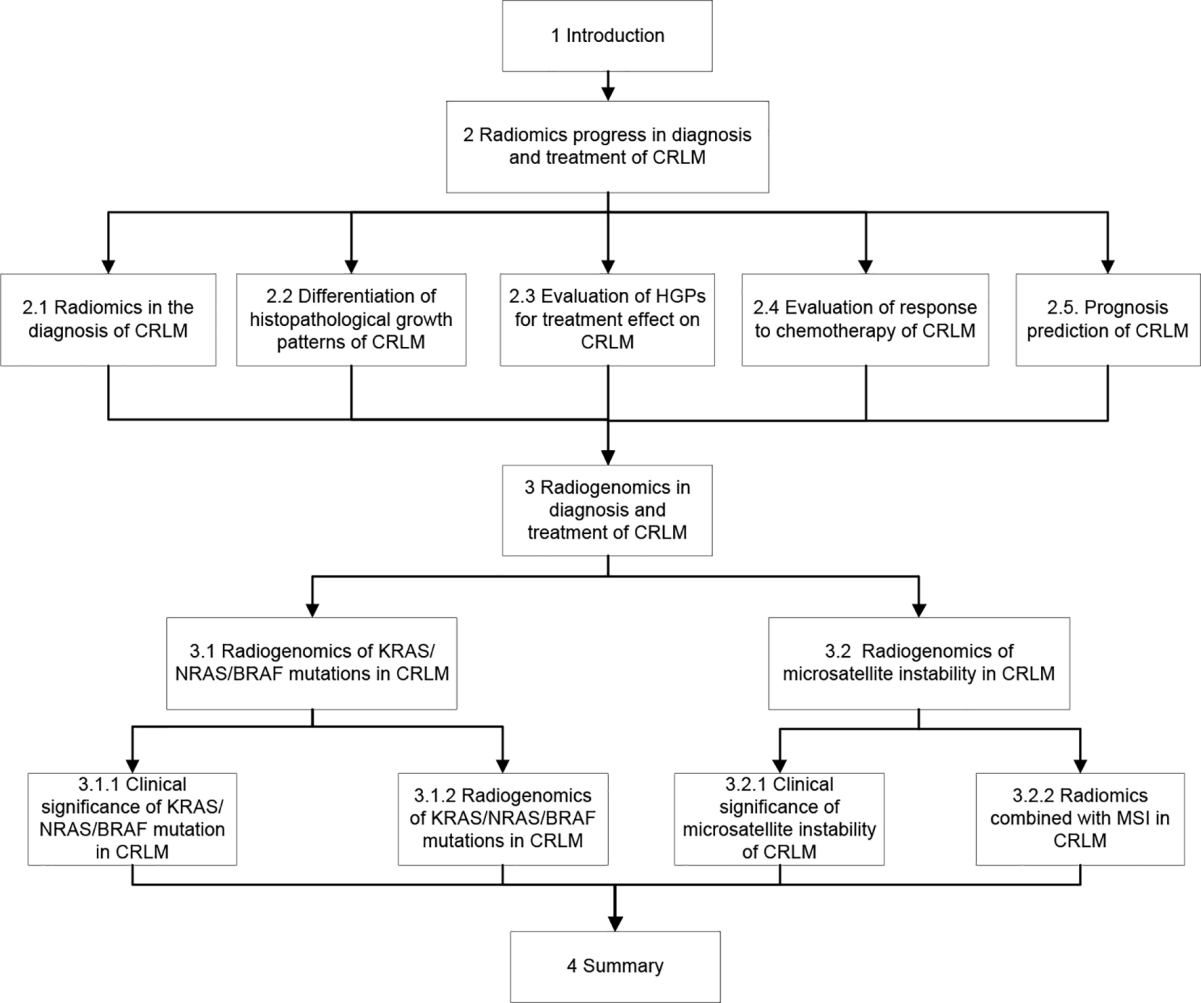
Flow chart of this paper.

In patients with CRC, one factor significantly affecting the prognosis is the proper management of colorectal cancer liver metastases (CRLM), and surgical treatment stands for the only opportunity of long-term survival. The 5-year survival rate of CRC patients with complete resection of liver metastases has been reported to be approximately 30% higher than that without appropriate treatment of the liver metastases (16). Therefore, one of the keys to improving the prognosis of CRC patients is to detect liver metastases for initiating appropriate treatment as soon as possible. Currently, few studies have been performed on radiomics or radiogenomics of CRLM, and this review focused on the radiomics and radiogenomic features of CRLM, trying to facilitate early detection and appropriate treatment of CRLM besides evaluation of its genetic factors and response to treatment for improving the prognosis. The flow chart of the content of this paper is shown in **Figure 2**.

## 2 Radiomics progress in the diagnosis and treatment of CRLM

In recent years, the field of medical image analysis has developed rapidly, and the development of pattern recognition tools has promoted fast progress of quantitative feature extraction. By extracting a great deal of quantitative features from medical imaging data, radiomics can be used to analyze image information in detail. Compared with traditional approaches of imaging diagnosis, it can significantly improve tumor diagnosis, grading and staging, evaluation of responses to chemotherapy, and prognosis prediction (15, 17, 18), providing professional guidance for treatment planning.

### 2.1 Radiomics in the Diagnosis of CRLM

With the progress of imaging technology, conventional imaging approaches can effectively detect large and typical CRLM. However, due to the complexity of hepatic hemodynamics and differences of liver parenchymal background on imaging among patients, different imaging modalities perform differently in diagnosis of atypical or tiny liver metastases. It is hard to detect tiny or occult metastases by using the existing imaging approaches; however, identification of these lesions is crucial to early management and improved prognoses. Radiomics features, including entropy, texture and texture ratio, uniformity, and convolutional neural networks (CNNs), have been effectively applied for diagnosis of CRLM. In assessing the capability of whole-liver CT imaging texture analyses of hepatic parenchyma in distinguishing CRC patients with simultaneous hepatic metastasis (*n* = 10), heterochronous hepatic metastasis within 18 months after initial staging (*n* = 4), or no hepatic metastasis (*n* = 15), Rao et al. (19) found that the mean entropy of the whole liver was significantly (*p* < 0.05) higher in patients with synchronous metastases than those without hepatic metastases, whereas the mean uniformity of the whole liver was significantly (*p* < 0.05) lower in patients with synchronous metastases than those without liver metastases. This study indicated that texture evaluation of seemingly disease-free liver is promising to distinguish CRC patients with or without hepatic metastases. After analyzing the texture in non-enhanced CT imaging in seemingly non-diseased regions of liver for impact of hepatic texture by presence of malignant tumors in patients with CRC, Ganeshan et al. (20) found that the fine to medium texture ratio after imaging filtration was significantly (*p* < 0.05) different in seemingly non-diseased hepatic areas in patients with hepatic metastasis compared with those without liver metastasis (entropy, *p* = 0.0257) or those with extra-liver disease (uniformity, *p* = 0.0143). Imaging textures of entropy and uniformity have been found to be more advantageous to other features in the diagnosis of CRLM.

CNNs are able to generate useful characteristics from imaging data and have been proven to have high values in predicting oncological outcomes (5, 21–23). Lee et al. used CNNs to generate imaging features from the liver parenchyma in 2019 patients with stage I–III CRC for predicting metachronous liver metastasis based on preoperative abdominal CT imaging (5). They found that the radiomics model combining clinical variables with the top principal components of imaging had the greatest performance (mean AUC = 0.747) to predict 5-year metachronous liver metastasis compared with the model using clinical features only. Even though no hepatic metastasis was found during the initial colectomy, the radiomics features using the CNNs could be used to predict possible metachronous liver metastasis.

### 2.2 Differentiation of Histopathological Growth Patterns of CRLM

The heterogeneities of genetic, phenotypic, epigenetic, and morphological features inside and outside the CRLM lesion result in different responses to systemic treatment (24, 25). The histopathological growth pattern (HGP) is one such heterogeneity with corresponding microvasculatures. Based on the interface of cancerous cells with adjacent hepatic texture, CRLM has two primary kinds of HGPs: replacement and desmoplastic, with other uncommon kinds of mixed and pushing HGPs (26). The desmoplastic HGP is characterized by separation of the cancerous cells from the hepatic texture by a fibrous band with lymphocytic infiltration and sprouting angiogenesis in the microvasculature. In this pattern, the cancerous cells initiate a reaction similar to the healing of wounds: scar tissues are created with presence of inflammation and new blood vessels. In the replacement HGP, the cancerous cells constitute cellular plates that are in continuity with the hepatocytic plate, allowing the cancerous cells to displace hepatocytes and co-opt the sinusoidal blood vessels at the cancer–liver interface, without disturbing the hepatic stromal architecture or inducing sprouting angiogenesis (25, 27, 28). Desmoplastic metastases are frequently well or moderately differentiated, whereas replacement liver metastases are of poor differentiation, lacking immune reaction and secondary glandular structures (27, 29). The pushing HGP is less common with the hepatocyte plate being compressed and pushed away by the metastatic cancer cells, with no desmoplastic rim around the cancerous cells or direct contact of the cancerous cells with the hepatocytes.

The HGPs of CRLM can be effectively differentiated using multidetector CT-based radiomics and MRI-based radiomics (multi-habitat and multi-sequence) (25, 30). After studying 126 patients with CRLM lesions who had undergone abdominal contract-enhanced CT imaging followed by partial hepatectomy with histopathologically confirmed HGPs including desmoplastic HGP in 68 patients and replacement HGP in 58, Cheng et al. (30) found that the fused radiomics signature had the best predictive performance in differentiating replacement from desmoplastic HGPs (AUC of 0.926 and 0.939, respectively, in the training and validating set), with good discrimination demonstrated in the clinical-radiomics combined model (C-indices of 0.941 and 0.833, respectively, in the training and validating set). Han et al. (25) investigated MRI data of 182 resected CRLM lesions in chemotherapy-free patients including desmoplastic HGPs in 59 patients and replacement HGPs in 123, with the decision tree algorithm being used for radiomics modeling, fused radiomics model being reconstructed from combination of radiomics signatures of all sequences, and clinical and combined models being constructed *via* multivariate logistic regression analysis. They found that the fused radiomics model of tumor zone and the radiomics model of tumor–hepatic interface zone exhibited superior performance to any single sequence or the clinical model and that the radiomics model of tumor–liver interface zone was better than that of the tumor zone (AUC of 0.912 vs. 0.879). The combined model had good discriminating capability, with the AUC of nomogram being 0.971, 0.909, and 0.905, respectively, in the training, internal validating, and external validating set. Their study (25) revealed that MRI-based radiomics is capable of predicting the predominant CRLM HGPs as a potential biomarker for therapeutic strategy. Through analysis of the above studies, it was found that the combination model of radiomics and clinical information can show better discrimination ability than the single radiomics model.

### 2.3 Evaluation of HGPs for Treatment Effect on CRLM

Metastases are the major death cause in most patients with solid malignancies, and hepatic metastasis is the critical factor for survival of patients with advanced malignant tumors (27, 28). Histological presentations of liver metastases are heterogeneous and reflected by different HGPs that affect clinical outcomes. The desmoplastic HGP is a positive prognostic biomarker while the replacement HGP is a negative one (27). A retrospective study enrolling 732 patients found that the exclusive desmoplastic growth serves as a positive prognostic marker for patients with CRLM, which is not matched by any other factors evaluated (31). In this study, 19% of patients without chemotherapy (*n* = 367) had desmoplastic growth in the whole tumor–hepatic interface and were independently associated with 50% 5-year survival rate without progression (hazard ratio or HR: 0.54, *p* = 0.001) and 78% 5-year overall survival (HR: 0.39, *p* < 0.001). CRLM lesions with this kind of HGP are more suitable for regional metastases-directed treatment. On the contrary, replacement HGP is linked to poor pathological responses, with the presence of a large proportion of cancerous cells after chemotherapy, and bad imaging reaction on CT in patients with primary chemotherapy and anti-angiogenesis therapy before surgery for CRLM (32). This type of HGP occurs more often in new hepatic metastatic lesions that grow even during systemic therapy. The replacement HGP indicates not only worse overall and progression-free survival (31, 33, 34), but also resistance to systemic therapy in patients with CRLM (32). A possible reason for the resistance to systemic therapy of the replacement type of HGP is vessel co-option, which serves as an approach of continuous blood supply when the vascular endothelial growth factor is inhibited by treatment (35). Moreover, different HGPs have varied immune phenotypes that contribute to varied responses to immune therapy. Evidence has indicated that tumors with limited numbers of infiltrated T cells are in essence frequently resistant to immune therapy (36). Vascular co-opting hepatic lesions of metastasis usually have low infiltration of immune cells or inflammatory cells as demonstrated in lesions with the replacement type of HGP in contrast to those with desmoplastic HGP which are frequently surrounded by a lot of lymphocytes in the dense rime (29, 37). Thus, the types of HGPs differentiated using the multidetector CT-based radiomics and MRI-based radiomics (25, 30) may indicate the prognosis of patients with relevant types of HGP in CRLM lesions. In studying the HGP types of CRLM using MRI-based radiomics in comparison with the histopathological types, Han et al. (25) found that more tissue types were presented in the desmoplastic HGP lesion of CRLM, including inflammatory, fibrosis, tumor, and hepatic cells, indicating greater heterogeneity than lesions of replacement HGP. Replacement and desmoplastic HGPs may be able to predict responses to bevacizumab and long-term prognosis. Galjart et al. have convincingly demonstrated that patients with CRLM and an exclusive desmoplastic HGP (100% of the tumor–hepatic interface) undergoing partial hepatectomy have outstandingly good outcomes (31).

### 2.4 Evaluation of Response to Chemotherapy of CRLM

In CRLM patients, less than 30% were initially resectable (38). In some patients, the metastatic foci, which could not be removed, might disappear on imaging after appropriate therapy, but some metastases could still be detected during radical surgery. Because radiomics can explore subtle changes of tumor and liver texture before and after treatment, it can be used to evaluate the response of CRLM lesions to chemotherapy (39–48). The CRLM lesion uniformity, entropy, homogeneity (variance and angular second moment), gray-tone difference, matrix contrast and shape, skewness, narrowed standard deviation, mean attenuation, density of major hepatic lesion, and histogram parameters for apparent diffusion coefficient maps have all be used to predict responses to chemotherapy. Good responses have been associated with decreased entropy, increased uniformity, higher variance, lower angular second moment, lower baseline skewness value, narrowed standard deviation, high mean attenuation, mean values of histogram parameters for apparent diffusion coefficient maps, and high baseline density of dominant hepatic lesions.

The entropy of CRLM lesions had been reported to decrease in patients with good responses while the uniformity increased after chemotherapy (entropy: −5.13 in good responding patients and +1.27 in non-responding patients, OR = 1.34; uniformity: +30.84 vs. −0.44, respectively, OR = 0.95) (45). However, a higher entropy had also been associated slightly with therapeutic success (6.65 ± 0.26 in patients with good responses vs. 6.51 ± 0.34 in non-responding patients, *P* = 0.08) (41), and a low baseline uniformity was related to a good response (cutoff ≥ 0.42; OR = 20, 95%CI = 1.85–217.4) (46). Two measures for homogeneity of lower angular second moment and a higher variance had been demonstrated to associate with good responding CRLM lesions rather than non-responding lesions on T2 MRI imaging, with the variance of 446.07 ± 329.60 in patients with good responses vs. 210.23 ± 183.39 in non-responding patients (*p* < 0.001) and the angular second moment of 0.96 ± 0.02 vs. 0.98 ± 0.01, respectively (*p* < 0.001).

After investigating therapeutic radiomics features for predicting tumor sensitivity in 667 patients with CRLM to 5-fluorouracil, irinotecan, and folinic acid alone or combined with cetuximab, Dercle et al. (42) found that the radiomics response signature outperformed known biomarkers of the KRAS mutation status and tumor contraction rate in the early prediction of therapeutic sensitivity and for guiding decisions of cetuximab therapy. In evaluating the significance of pre-treatment CT texture analyses for predicting treatment responses in 82 patients with CRLM after combined targeting chemotherapy, Zhang et al. (49) found significant (*p* < 0.05) differences in Entropy, Energy, Variance, Standard deviation, Quantile 95, and sumEntropy between the response and non-response groups in pre-treatment lesions. Lesions with higher Entropy, lower Energy, higher Variance, higher Standard Deviation, and higher sumEntropy seemed to indicate a better therapeutic response. Good diagnostic efficiency was obtained when sumEntropy > 0.867, with a sensitivity of 60.5% and a specificity of 79.5%. Radiomics texture indexes originating from basic CT imaging data of CRLM lesions had the potential capability of imaging biomarkers for predicting cancer response to targeted chemotherapy. By comparing the image features before and after diagnosis and treatment, we found statistically significant radiomics features, such as Entropy, Energy, Variance, Standard deviation, and Quantile, which can all be used to evaluate the remission effect of drugs on CRLM lesions. In the future, these radiomics features can be used clinically as a relatively cheap and noninvasive monitoring means for patients with CRLM or other malignancies.

Most of the reported studies on radiomics are based on CT images, and radiomics features from MRI images can also be used to predict the treatment effect on liver metastases. In order to determine the predictive value of pre-treated MR texture features of CRLM lesions for therapeutic response to chemotherapy, Zhang et al. (48) extracted five histogram features (variance, mean, kurtosis, skewness, and entropy) and five co-occurrence matrix features of gray level (GLCM; entropy), angular second moment, correlation, inverse difference moment, and contrast) from whole liver MRI T2WI data of 26 patients with CRLM before chemotherapy. After careful evaluation, a higher variance, contrast, entropy, entropy, a lower angular second moment, correlation, and inverse difference moment were revealed to significantly (*p* < 0.05) independently associate with good responses to chemotherapy (AUCs 0.602–0.784). Multivariable logistic regression demonstrated that variance (*p* < 0.001) and angular second moment (*p* = 0.001) remained predictive parameters to distinguish responding from non-responding tumors, with the highest AUC of 0.814 (48).

### 2.5 Prognosis Prediction of CRLM

After active surgical resection, radiofrequency, and chemotherapeutic targeted therapy, some patients with CRLM can achieve a high-quality survival of up to 10 years, whereas others only obtain a short tumor-free survival. Individual differences make the application of personalized treatment strategy particularly important, and identifying risk factors allows clinicians to develop surveillance strategies for patients who are at a higher risk of recurrence. Researchers all over the world have proposed many scoring systems for grading and predicting prognosis of CRLM patients with different tumor loads (17–19), but the ultimate effects on prognosis may be quite different. The radiomics features of heterogeneity, homogeneity, uniformity, Graytone difference matrix contrast, spatial heterogeneity, entropy, texture, and gray level size zone matrix have been used to evaluate the prognoses of patients with CRLM.

Radiomics features have been used to predict the survival of patients with CRLM who have undergone chemotherapy or hepatic surgery because radiomics can assess subtle liver texture differences on different images (40–43, 46, 47, 50–53). An association had been revealed between CRLM heterogeneity/homogeneity and survival. Patients with a greater uniformity of CRLM on CT imaging (cutoff value ≥ 0.42 with a relative risk of 6.94 for overall survival and a relative risk of 5.05 for progression-free survival) had been reported to have poor overall survival and progression-free survival (46). A shorter overall survival had also been demonstrated to associate with metastatic homogeneity on CT imaging (HR: 1.5 × 10^20^–1.3 × 10^49^) (40). After comparing with before chemotherapy, a radiomic signature based on two heterogeneity features, Graytone Difference Matrix contrast and spatial heterogeneity, had been related to overall survival (HR = 44.3 for patients with superior image quality; HR = 6.5 for patients with conventional image quality) (42), with the radiomic signature having a better value in predicting survival than the 8-week tumor shrinkage or KRAS-mutational status assessed in accordance with the RECIST criteria (AUC 0.80 vs. 0.67 for KRAS and 0.75 for RECIST, *p* < 0.001) in the validation setting. The CRLM heterogeneity at 18F-FDG PET/CT was also confirmed to be a predictor of shorter overall survival (HR 4.29) at multivariant analysis (51), and a model constructed with numbers of metastases, histogram uniformity, and metabolic cancer volume was constructed to predict shorter event-free survival (HR 3.20, *p* < 0.001) (51).

Entropy of the metastatic lesions had been associated with the prognosis of patients with CRLM (40, 41, 50). It had been reported that the overall survival was in a positive correlation with the entropy of CRLM [HR: 0.16–0.63 (40), and HR = 0.65, 95% CI = 0.44–0.95 (50)]. The value of entropy ratio between CRLM and liver texture had also been demonstrated to relate to the prognosis, with a negative correlation between the value and overall survival (HR 1.9) (41). After studying the tumor and liver texture on CT portal venous-phase images in 230 patients with CRLM (120 in the training and 110 in the validation group) before and 2 months after chemotherapy, Dohan et al. (43) established a predictive model of efficacy after 6 months of chemotherapy, which is as effective as the RECIST1.1 evaluation criteria for solid tumors. The radiomic signature with the combination of decreases in sum of target liver lesions, density, and texture analyses of dominant liver lesion at baseline and 2-month CT imaging data could predict the overall survival and detect tumors with good responses better than the RECIST1.1 criteria for CRLM treated by bevacizumab and FOLFIRI as first-line medicines.

Other radiomics features have also been related to the survival. The combination of CRLM correlation and contrast into a single texture parameter had been reported to associate with overall survival (HR 2.35) (53). One texture analysis score combining three features of high baseline density of dominant hepatic lesion, reduction in kurtosis, and decrease in the sum of target hepatic lesions assessed 2 months after chemotherapy had been demonstrated to strongly associate with overall survival (SPECTRA score >0.02 vs. ≤0.02, with the HR of 2.82 in the training set and 2.07 in the validating set) (43). Radiomic evaluation score 2 months later had the same prediction value of prognosis as the RECIST criteria following chemotherapy for 6 months. In the gray level size zone matrix, the small area emphasis (positive parameter of prognosis, HR 0.62) and the minimal pixel value (negative parameter, HR 1.66) had been revealed to be related to progression-free survival (52).

In addition to the above mentioned radiomics features, CRLM density on CT imaging (46), ShapeSI4 (in a radiomic signature) (42), standard deviation (40), future hepatic residual energy and entropy combined as a single linear predictor (53), and AUC of volume histograms at PET-CT (47) have also been reported to associate with the overall survival.

## 3 Radiogenomics in Diagnosis and Treatment of CRLM

Radiogenomics can be used to discover the radiomics features that reflect gene expression or polymorphism for further understanding the occurrence and development of diseases (54). Radiogenomics promises to understand gene expression of diseases through noninvasive and conventional imaging methods. With continuous progress of the technology, radiogenomics has been widely studied in systemic diseases in recent years. Many scholars have reported a correlation between radiomics features and EGFR (epidermal growth factor receptor) mutation (55–57) or ALK (anaplastic lymphoma kinase) rearrangement of lung cancer (58, 59). In detection and management of breast cancers, many researchers have found that breast cancer is associated with radiomics features at the gene sequence level (60), gene expression level (61), and molecular subtype level (62). Marigliano et al. (63) analyzed multiphase CT images (arterial phase, portal-venous phase, and urinary tract phase) of 20 patients with clear cell renal cancer and found that the radiogenomics data derived from these images were well correlated with expressions of some microRNAs (miR-185-5p, miR-21-5p, miR-210-3p, miR-221-3p, and miR-145-5p), especially between entropy and miR-21-5p. Similarly, progress has also been made in radiogenomics for prostate cancer (64).

Currently, there are only limited studies on radiogenomics of tumors involving the liver. Segal et al. were the first in 2007 to explore the correlation of gene expression pattern of a hepatocellular carcinoma with the imaging features, identifying 32 image characteristics from enhanced CT imaging of three phases to be correlated to the expression degrees of 116 genetic biomarkers among 6,732 genes confirmed by microarray analysis (65). However, only three imaging features on average were required to catch expression variations of any genetic marker, and the use of 28 image features combined could explain variations of all 116 genetic markers (65). Moreover, it was found that the genes in some particular molecular profiles had common physiological function, including cellular proliferation and hepatic enzyme syntheses, which could correlate to specific imaging characteristics. Thus, two image features, presence of arteries and absence of low-density halos, were found to correlate with “venous invasion signatures”, which are image patterns to predict microscopic venous invasion and OS (65). Kuo et al. (66) also conducted radiogenomic analysis to identify imaging traits in hepatocellular carcinomas, which were related to a genetic expression profile of 61 genes to detect tumor responses to doxorubicin. The enhanced CT imaging data of 30 hepatocellular carcinomas had been studied for six image features, which were found to correlate with the microarray of 18,000 genes.

CRC is a heterogeneous tumor, and its occurrence and development are affected by a variety of factors. Lifestyle habits such as high-fat diet are important risk factors to increase the incidence of CRC (67). Besides external factors, intrinsic genetic factors also affect the occurrence and development of CRC (68). Knowing the status of gene mutation in CRC can effectively provide guidelines for clinical treatment and prognosis evaluation, thus formulating a recurrence surveillance strategy for patients (69).

### 3.1 Radiogenomics of KRAS/NRAS/BRAF Mutations in CRLM

#### 3.1.1 Clinical Significance of KRAS/NRAS/BRAF Mutation in CRLM

The RAS/RAF/MEK/extracellular signal-regulated kinase signaling cascade is referred to as the pathway of mitogen-activated protein kinase (MAPK), which controls cellular differentiation, proliferation, angiogenesis, migration, and survival. Dysregulation of the pathway constitutes the bases for tumorigenesis (70). This pathway consists of RAS small guanidine triphosphatases (GTPase) and can activate the family proteins of RAF (ARAF, CRAF, and BRAF). Abnormal activation or signaling of the MAPK pathway had been demonstrated in many tumors, including CRC, through some distinctive mechanisms, like mutations in BRAF and RAS (70), which most frequently occur in human neoplasms.

KRAS, NRAS, and HRAS are the RAS oncogenes to encode a family of GTP-adjusted switches and can repeatedly mutate in human cancers (71). Once activated, these genes will cause pleiotropic effects in cells, leading to cellular differentiation, proliferation, and survival. KRAS mutations take up approximately 85% of mutations in the RAS gene in human malignancies, NRAS accounts for approximately 15%, and HRAS accounts for below 1% (72). In CRC, RAS mutations primarily take place in the KRAS gene, and approximately 45% of metastatic CRCs contain activated KRAS mutations (73). NRAS mutation happens in 2%–7% patients with metastatic CRC (71). KRAS gene mutations are related to right-sided colonic cancers, but NRAS gene mutation is related to left-sided primary malignancies and female gender, indicating distinctive biology for NRAS and KRAS mutant molecule subsets of metastatic CRC (74).

KRAS gene is related to the pathogenesis and progression of CRC, and mutation of this gene may cause resistance to EGFR inhibitors and poor tumor response to molecular targeted drugs (75, 76). De Macedo et al. (77) studied the DNA of primary tumor and metastatic tissue in 102 cases of CRLM and found that the KRAS gene was highly homogeneous across the primary CRC cancer areas and consistent in the original cancer lesion with the metastatic tissues in the same person. KRAS mutation is an independent risk factor for the prognosis of patients with CRC (78). Therefore, understanding the KRAS mutation rate in patients with CRC will help treatment planning and prognosis evaluation.

NRAS defines a group of molecules with different clinical features from KRAS-mutant and wild-type metastatic CRC (71). NRAS gene mutation can cause disordered malignant proliferation and promote metastasis (71), thus associating with worse survival and outcomes than KRAS-mutant or wild-type metastatic CRC. Activating mutations in NRAS take place in 30% of cases with skin melanoma, and BRAF mutation happens at a high incidence in these malignancies (74). BRAF or NRAS gene mutation is related to poor survival of metastatic melanoma patients. However, BRAF mutation is reciprocally exclusive with melanoma NRAS mutation and with CRC KRAS mutation.

BRAF mutations take place in 7% of cancers, and approximately 8%–12% of metastatic CRC cases contain BRAF mutations (79). BRAF gene mutation can cause poor drug effect and worse prognosis, and reduce the effect of cancer cell apoptosis, thus aggravating the condition of patients with cancers. Some studies (80) found that the mutation rate of the BRAF gene is higher in patients with lower tumor differentiation.

#### 3.1.2 Radiogenomics of KRAS/NRAS/BRAF Mutations in CRLM

Yang et al. (81) studied 346 radiomic features extracted from portal venous-phase CT imaging data of primary tumors and KRAS/NRAS/BRAF gene mutation in 117 patients with CRC, including 61 cases in the training and 56 in the verification group before treatment. The support vector machine methods and RELIEFF were constructed to choose important features and establish the radiomic features. It was found that the radiomic signature was significantly associated with the KRAS/NRAS/BRAF mutation (*p* < 0.001), with the AUC, sensitivity, and specificity for predicting KRAS/NRAS/BRAF mutation as 0.869, 0.757, and 0.833 in the primary group, and 0.829, 0.686, and 0.857 in the validation group, respectively.

Lubner et al. (50) investigated tumor texture analysis on single CRLM lesion on contrast-enhanced CT imaging in 77 patients before treatment. It was found that entropy (spatial scaling factor or SSF 4, *p* = 0.007), mean positive pixels (SSF 3, *p* = 0.002), and standard deviation (SSF 3, *p* = 0.004) of medium filtration were significantly associated with the tumor stage. Skewness was found to negatively associate with KRAS mutations (*p* = 0.02), whereas the coarse filtration entropy was significantly (*p* = 0.03) associated with survival (HR for death 0.65). Therefore, radiogenomics is expected to understand the gene expression profile of the disease through noninvasive and routine imaging examination and may be a breakthrough in the diagnosis, treatment, disease monitor, and prognosis evaluation of CRC and CRLM.

### 3.2 Radiogenomics of Microsatellite Instability in CRLM

#### 3.2.1 Clinical Significance of Microsatellite Instability of CRLM

Some kinds of genomic instability are able to drive the initiation and development of CRC. The most common type is chromosomal instability, which is found in 85% of CRC, and another is microsatellite instability (MSI) which occurs in 15% patients with CRC. MSI tumors are a subset of CRC characterized by malfunction of mismatch repair genes (MMR), which can cause failure to repair errors in short tandem repetitive DNA sequences known as microsatellites (82, 83). In the microsatellite sequences, the DNA replication stability is poor and is prone to mismatches. MSI is caused by lack of DNA mismatch repair (MMR) system, arising from germline mutations in the MMR gene, which is prone to the Lynch syndrome, or from epigenetic inactivation of MLH1 in sporadic malignancies. Approximately 5% metastatic CRCs showed MSI or deficient MMR, and sporadic CRC patients with MSI were often related to BRAFV600E mutation *via* its association with CpG methylator phenotype (84).

High-frequency MSI (MSI-H) refers to the occurrence of MSI at two or more sites; low-frequency MSI (MSI-L) is MSI occurring only at one site; microsatellite stability (MSS) indicates MSI, which does not occur at any site (85, 86). MSI has a guiding role in predicting the malignant degree and pathogenesis of tumor, and can also provide direction for clinical selection of treatment plan and prognosis evaluation. Studies have shown that MSI-H can be used as a biomarker to guide clinical immunotherapy for CRLM patients (83, 84, 87). Through transformation therapy of immune drugs, it is possible to remove the metastatic foci so as to further improve survival and quality of life for cancer patients.

#### 3.2.2 Radiomics Combined With MSI in CRLM

Understanding the MSI status is necessary because CRC tissues with MSI have specific biological behavior and may indicate better prognoses and benefit from immunotherapy or resistance to fluorouracil treatment (88). However, the approaches for evaluating MSI status using polymerase chain reaction and immunohistochemistry are performed on pathological tissues from invasive biopsies or surgeries and have not been extensively applied. It is therefore necessary to develop non-invasive and cost-effective methods to predict the MSI status and guide further therapeutic strategies. By extracting 254 radiomics features of intensity from CT imaging of the CRC cancer region in combination with clinical features in 198 patients including 134 patients with microsatellite stable tumors and 64with MSI tumors, Golia Pernicka et al. (89) were able to develop three prediction models with clinical features only, radiomic features only, and combination of radiomic and clinical features. The combined radiomics model outperformed the other two models in predicting MSI, with the AUC of 0.80 and 0.79 for the training and testing set, respectively (specificity 96.8% and 92.5%, respectively).

Fan et al. studied 119 patients with stage II CRC confirmed pathologically, known MSI status, and preoperative enhanced CT images for extracting radiomics features (90). In their study, the radiomics features were obtained from the portal-vein phase CT imaging data of segmented tissues of each complete primary cancer lesion with the Matrix Laboratory software while the radiomic signatures were constructed using the selection operator logistic regression and least absolute shrinkage model. Six radiomics and 11 clinical features were chosen for predicting the MSI status. The model combining both radiomic and clinical features achieved the overall best performance in predicting the MSI status than either the radiomics or clinical feature model alone, yielding the AUC, sensitivity, and specificity of 0.752, 0.663, and 0.841 for the combined model, 0.598, 0.371, and 0.825 for clinical model alone, and 0.688, 0.517, and 0.858 for radiomics model alone, respectively. Combined analyses of radiomic and clinical features improved the predictive efficacy and helped selecting appropriate patients for personalized therapy.

In exploring the value of radiomics analysis derived from dual-energy CT imaging to preoperatively evaluate the MSI status in CRC, Wu et al. (88) investigated 102 CRC patients with pathologically confirmed MSI status and selected nine top features to constitute the radiomic model. They found that radiomic analyses of iodine-based material decomposition imaging data with dual-energy CT has a great capability to predict the MSI status in patients with CRC, with the AUC, accuracy, sensitivity, and specificity of 0.961, 0.875, 1.000, and 0.812 in the training set, and 0.875, 0.788, 0.909, and 0.727 in the testing set, respectively. Good clinical application and calibration were demonstrated with the decision curve and calibration analyses, respectively.

Although there is consistency between CRC MSI status and liver metastasis, there were currently no correlation studies between MSI status and radiomics of liver metastasis.

## 4 Summary

In the diagnosis, treatment, monitor of disease progression, and prognosis of CRLM, thousands of radiomics features can be extracted, such as image intensity features, high-order features, texture features, and shape features. Due to the lack of unified standards at present, different research teams choose different radiomics features in the selection of features. Through review of published studies in the literature, it is found that the most widely used radiomics features include entropy, uniformity, variance, and skewness. At present, the unity of the results is relatively poor, but all these results show the feasibility and significance of the application of radiomics and radiogenomics in the diagnosis, treatment, monitor of disease progression, and prognosis of CRLM.

Radiomics and radiogenomics can be widely used in clinical medicine research with noninvasiveness and low cost. However, as a new field, it is still in its infancy, with many limitations. For example, the research data for radiomics mostly come from small samples and single centers, whereas some big data from multicenters are different because of use of different scanning equipment and scanning conditions. Moreover, imaging delineation segmentation approaches may also differ from center to center or from study to study. Future development and research in radiomics and radiogenomics will have to solve these issues for better outcomes.

As an innovative arena in medical imaging, radiomics and radiogenomics can be used to identify pathological process, reveal the underlying pathophysiological mechanisms through medical imaging and clinical data, and identify hidden imaging patterns that can be used to predict tumor biological behavior and patients’ prognoses, providing efficient prediction of responses to chemotherapy and survival in addition to accurate and early prediction compared to standard biomarkers. Continuous surveillance of the radiomics and radiogenomics biomarkers will provide adequate information to monitor cancer recurrence and individualized treatment to the constantly changing genome of cancer. The current research results in radiomics and radiogenomics of CRLM warrant further exploration into wider application in other fields.

## Author Contributions

Study design: X-PY and B-LG. Data collection: YW and L-YM. Supervision: L-YM. Writing of the original version of article: YW. Revision of the original version: B-LG. All authors contributed to the article and approved the submitted version.

## Funding

This study is funded by Hebei Natural Science Foundation Project (H20212017), Project of Hebei Provincial Department of finance (361007), and Medical Discipline Cultivation Project of Hebei University (2020b05).

## Conflict of Interest

The authors declare that the research was conducted in the absence of any commercial or financial relationships that could be construed as a potential conflict of interest.

## Publisher’s Note

All claims expressed in this article are solely those of the authors and do not necessarily represent those of their affiliated organizations, or those of the publisher, the editors and the reviewers. Any product that may be evaluated in this article, or claim that may be made by its manufacturer, is not guaranteed or endorsed by the publisher.
